# Lifestyle Behaviors of African American Breast Cancer Survivors: A Sisters Network, Inc. Study

**DOI:** 10.1371/journal.pone.0061854

**Published:** 2013-04-23

**Authors:** Raheem J. Paxton, Wendell C. Taylor, Shine Chang, Kerry S. Courneya, Lovell A. Jones

**Affiliations:** 1 Dorothy I. Height Center for Health Equity & Evaluation Research at the University of Houston/The University of Texas MD Anderson Cancer Center, Houston, Texas, United States of America; 2 Division of Health Promotion and Behavioral Sciences at The University of Texas Health Science Center at Houston, Houston, Texas, United States of America; 3 Department of Epidemiology at The University of Texas MD Anderson Cancer Center, Houston, Texas, United States of America; 4 Faculty of Department Physical Education and Recreation, University of Alberta, Edmonton, Canada; University of Porto, Portugal

## Abstract

**Introduction:**

African American breast cancer survivors experience poor cancer outcomes that may, in part, be remedied by healthy lifestyle choices. Few studies have evaluated the health and lifestyle behaviors of this population. The purpose of this study was to characterize the health and lifestyle habits of African American breast cancer survivors and evaluate the socio-demographic and medical correlates of these behaviors.

**Methods:**

A total of 470 African American breast cancer survivors (mean age = 54 years) participated in an online survey. All participants completed measures assessing medical and demographic characteristics, physical activity, and sedentary behavior. Chi-square tests for association, nonparametric tests, and logistic regression models were used to assess associations. All statistical tests were two sided.

**Results:**

Almost half (47%) of the women met the current guidelines for physical activity, almost half (47%) were obese, and many reported having high blood pressure (53%) or diabetes (21%). The prevalence of high blood pressure, diabetes, and high cholesterol increased by age (P<0.001), and obese women had a higher prevalence of high blood pressure (63% vs. 44%) and diabetes (21% vs. 12%) than did non-obese women (all P<0.05). Obese women participated in significantly fewer total minutes of physical activity per week (100 minutes/week) than did non-obese women (150 minutes/week; P<0.05). The number of comorbid conditions was associated with increased odds for physical inactivity (odds ratio = 1.40) and obesity (odds ratio = 2.22).

**Conclusion:**

Many African American breast cancer survivors had chronic conditions that may be exacerbated by poor lifestyle choices. Our results also provide evidence that healthy lifestyle interventions among obese African American breast cancer survivors are urgently needed.

## Introduction

Scientific advancements in cancer treatment during the past 30 years have resulted in improvements in cancer-specific and overall survival rates [1]. For example, 5-year in situ breast cancer survival rates exceed 98% [2]. Despite the overall improvement in the health and well-being of cancer survivors, similar improvements have not been observed among African American (AA) women [3]. Five-year relative survival rates among AA breast cancer survivors are approximately 23% lower than those of non-Hispanic white women, and AA women have higher rates of comorbid conditions such as cardiovascular disease and diabetes [4], [5], [6], [7]. Some researchers have speculated that many AA breast cancer survivors fail to engage in preventive health behaviors (e.g., cancer screening, weight management, physical activity [PA], healthy diet) [3], thereby increasing their vulnerability to comorbid conditions and cancer recurrence after primary cancer treatment [8].

PA is associated with several benefits throughout the cancer continuum, including improvements in cancer-related symptoms (e.g., fatigue, nausea, and pain), functional status, body mass index (BMI), and mood [9], [10], [11], [12]. In addition, a recent meta-analysis reported that post-diagnosis PA is associated with a ∼24% reduction in breast cancer recurrence and a ∼50% reduction in breast cancer deaths [13]. Conversely, sedentary behavior (SB; e.g., sitting, lying, lounging) is an independent risk factor for many chronic diseases, including diabetes and cardiovascular disease, as well as premature mortality [14], [15], [16], [17]. Recent studies of cancer survivors have shown that prolonged periods of SB are associated with larger waist circumferences [18]. Both abdominal obesity and general obesity have been shown to be causal factors in cancer initiation, development, and metastasis [19], [20], [21]. Importantly, AA breast cancer survivors are generally more sedentary, fail to meet current guidelines for PA, and have higher rates of general and abdominal obesity, thereby elevating their risk for cancer recurrence and prolonged disability [22], [23].

Few studies have reported on the health behaviors of AA breast cancer survivors, despite their increased vulnerability to poor health outcomes. Studies that provide data on the health behaviors of these women may help identify the factors that contribute to poor outcomes in this population. Descriptive studies are a necessary first step in filling the void and documenting the health and well-being of AA breast cancer survivors. Therefore, the purpose of this study was to (a) characterize the health behaviors of a sample of AA breast cancer survivors, (b) determine whether these behaviors differ by age group or obesity status, and (c) identify the determinants of lifestyle behaviors in this population.

## Methods and Materials

### Study Population

AA breast cancer survivors aged 18–70 years were identified through Sisters Network, Inc., the largest AA breast cancer survivorship organization in the United States. These women were recruited via solicitation emails about our survey and via anonymous survey links on social media sites and Sisters Network blog sites between April and July 2012. All surveys were completed using Survey Monkey, a web-based platform that allows investigators to create surveys, perform routine updates, and manage survey responses. Participants were eliminated from the final analyses if they were not breast cancer survivors, were not AA, or reported being diagnosed at younger than 18 years. The project was approved by the Institutional Review Board at The University of Texas MD Anderson Cancer Center prior to data collection, and a consent form was included on the initial survey web page. The Institutional Review Board approved all procedures, including the development of the web-based survey and the use of a passive consent form, before the survey was administered.

### Measures

Guidelines for PA were assessed via a self-administered instrument designed for the Women’s Health Initiative [24]. PA was calculated separately for light (metabolic equivalent task [MET] level <3.0), moderate (MET level 3.0–5.9), and vigorous (MET level ≥6.0) activities. A variable was also created for moderate-to-vigorous PA (MET level ≥3.0), which was then used to create a dichotomous variable (“meeting or not meeting PA guidelines”) based on a cutoff of 10.0 MET hours per week, which equaled approximately 150 minutes per week of moderate-paced walking or the equivalent of other exercise durations and intensities. The cutoff used in this study was consistent with the current recommendations of the Centers for Disease Control for PA [25] and has been validated in previous studies [26], [27].

#### Television-viewing time

Time spent watching television or videos was reported by participants separately for weekdays and weekend days during the previous week. Total television time was calculated as the sum of the time participants watched television on weekdays and weekend days. This measure has been shown to have reasonable reliability and validity for estimating television-viewing time in adults [28]. Although no current guidelines for SB exist, previous studies have indicated that television viewing in excess of 2 hours per day is associated with significant health effects [14], [29], [30], [31]. In view of the elevated risk for chronic diseases at 2 hours of reported television viewing, we created a variable to classify women with an average weekly television viewing time of 14 hours or less or greater than 14 hours.

#### Obesity status

The study participants’ self-reported height and weight were used to compute their BMI. BMI was computed in a standard manner: weight in kilograms was divided by height in meters squared (kg/m^2^). BMI risk categories were created to distinguish non-obese women from obese women and healthy women from normal/overweight (BMI<30 kg/m^2^) and obese (BMI≥30.0 kg/m^2^) women.

#### Socio-demographic and medical data

All socio-demographic and medical data were self-reported by participants. We collected data on the following variables: current age, race/ethnicity, marital status, education, treatment, smoking status, alcohol intake, time since diagnosis, disease stage at diagnosis, and comorbid conditions. Age was categorized to for an age group variable and we also summed the number of chronic conditions (e.g., cardiovascular disease, blood sugar/diabetes, digestive disorders, arthritis, and osteoporosis) that were self-reported.

### Statistical Analysis

Means and frequencies were used to characterize the study participants. Mean differences in continuous indicators were assessed with paired *t* tests. In the event that the distributions of the continuous variables were skewed or non-normal, we used a Wilcoxon rank-sum test to assess differences. Differences in categorical variables were assessed with a chi-square test of associations. We then used bivariate logistic regression models followed by multivariable forward stepwise logistic regression models to assess the associations of the medical and socio-demographic characteristics with the outcome variables (i.e., not meeting requirements for PA, excessive television viewing, and obesity status). In the stepwise regression model, the variables were progressively entered into the model if their significance level was at least 0.2 and were allowed to stay in the model if the significance remained at least 0.1 after adjusting for other variables. We opted to use this procedure to find the best possible model that fit the data. Indicators in multivariable (i.e., stepwise logistic regression analysis) models included age group, education, disease stage at diagnosis, years since diagnosis, smoking status, use of hormone replacement therapy, and income. In addition, PA and television viewing were included in models for obesity status and vice versa. The number of comorbid conditions was treated as a continuous variable in our models. Logistic regression models were reported in odds ratios (ORs) and 95% confidence intervals (CIs). These data were analyzed using the SAS Enterprise System version 4.2. All statistical tests were two-sided, and alpha <0.05 was considered statistically significant.

## Results

### Descriptive Characteristics

Of 760 people who initially visited the website, 525 visitors identified themselves as breast cancer survivors, and 307 completed the survey. The mean age of the survey participants was 54 years; the mean time since diagnosis was 7 years; and most participants were diagnosed with stage II disease. More than half had a least one comorbid condition, and half had high blood pressure. Fifty-three percent of the women report having high blood pressure, 28% had high cholesterol, and 21% had diabetes. Generally, younger women were married, were diagnosed at a later disease stage, and were more likely to have undergone chemotherapy (all P<0.05). In addition, a higher proportion of women 60 years and older reported having high blood pressure, cholesterol, and diabetes than did younger participants (all P<0.001). The highest proportion of participants with high blood pressure was observed in obese women, and the highest proportion of nonsmokers was observed in non-obese women (all P<0.05). Likewise, the number of comorbid conditions increased proportionally with age, and obese women reported having more comorbid conditions than did non-obese women (all P<0.01). The descriptive characteristics are reported in Table 1.

**Table 1:** Medical and demographic characteristics of AA breast cancer survivors.

Variable	Total	Age Groups			P-value	Obesity Status		P-value
		<50	50–59	≥60		Non-obese	Obese	
		n = 167	n = 160	n = 143		n = 250	n = 218	
**Mean Age (SD)**	53.8 (9.9)	43.4 (4.8)	54.3 (2.9)	65.5 (5.0)	–	53.9 (10.4)	53.8 (9.4)	0.941
**Mean Age at diagnosis, (SD)**	47.0 (9.1)	39.4 (5.4)	48.0 (5.7)	54.6 (8.7)		46.5 (9.4)	47.5 (8.7)	0.132
**Mean years since diagnosis, (SD)**	6.9 (6.3)	4.0 (3.6)	6.2 (5.4)	10.9 (7.5)		7.4 (6.7)	6.3 (5.8)	0.071
**Stage, n (%)**					<0.001			0.361
I	152 (35)	38 (24)	60 (39)	56 (43)		81 (36)	71 (34)	
II	188 (43)	66 (43)	62 (41)	60 (46)		101 (45)	87 (41)	
≥ III	95 (22)	51 (33)	30 (20)	14 (11)		43 (19)	52 (25)	
**Treatment, n (%)**								
Surgery	445 (95)	157 (94)	154 (96)	134 (94)	0.549	238 (95)	205 (94)	0.681
Chemotherapy	329 (72)	136 (81)	117 (73)	76 (53)	<0.001	166 (66)	161 (74)	0.087
Radiation	327 (71)	120 (72)	116 (73)	91 (64)	0.179	171 (68)	154 (70)	0.616
Hormone therapy	248 (48)	78 (47)	78 (49)	67 (47)	0.920	117 (47)	105 (48)	0.781
**Marital Status**					<0.001			0.185
Married	228 (49%)	83 (51%)	83 (52%)	62 (44%)		127 (51%)	101 (47%)	
**Education**					0.914			0.091
≤ high school	42 (9)	16 (10)	14 (9)	12 (8)		17 (7)	25 (12)	
Some College	184 (40)	70 (42)	58 (36)	57 (40)		92 (37)	92 (43)	
College Graduate	126 (27)	44 (27)	44 (28)	38 (27)		72 (29)	54 (25)	
Graduate degree	113 (24)	35 (21)	43 (27)	35 (25)		68 (27)	45 (21)	
**Select Comorbidities, n (%)**								
High blood pressure	248 (53)	56 (34)	92 (58)	100 (70)	<0.001	110 (44)	138 (63)	<0.001
Diabetes	76 (21)	8 (5)	34 (21)	34 (24)	<0.001	30 (12)	46 (21)	0.008
High Cholesterol	130 (28)	13 (8)	50 (31)	67 (47)	<0.001	63 (25)	67 (31)	0.214
**Number of comorbidities, m (SD)**	1.26 (1.14)	0.59 (0.81)	1.41 (1.08)	1.87 (1.15)	<0.001	1.10 (1.10)	1.44 (1.16)	<0.001
**Lifestyle Behaviors, n (%)**								
Never smoker	332 (72)	142 (86)	104 (66)	87 (62)	<0.001	184 (75)	148 (69)	0.013
Regular alcohol intake	62 (14)	140 (12)	134 (14)	119 (14)	0.873	202 (17)	190 (10)	0.074

P-values are based on non-parametric Kruskal-Wallis test (for age group) and Wilcoxon Rank Sum Test (for obesity). Estimate for moderate, strenuous, and walking are expressed in minutes/week.

### Lifestyle Characteristics

Self-reported television viewing and PA are reported in Table 2. A total of 53% were not meeting current guidelines for PA, 47% were obese, and 43% sat more than 2 hours per day while watching television. The total television-viewing time ranged from 7.2 to 21.2 hours per week, and the differences in television-viewing time according to age or weight status were not significant (all P>0.05). Leisure time moderate-to-vigorous PA ranged between 40 and 250 minutes per week, and the duration did not differ according to age. However, non-obese women reported significantly more total minutes of exercise per week and more minutes of moderate-intensity PA and walking than did obese women (P<0.05).

**Table 2:** Physical activity and sedentary habits of AA breast cancer survivors.

Variable	Total		Age Group			P-value	Obesity Status		p-value
			<50	50–59	≥60		Non-obese	Obese	
			n = 167	n = 160	n = 143		n = 250	n = 218	
**Sitting in hours/week in Median (25%, 75%)**									
TV									
Total	12.2 (7.2, 21.2)		11.7 (8.2, 20.2)	10.5 (6.2, 20)	13.2 (7.9, 24)	0.159	11.3 (7.2, 21.1)	12.2 (7.2, 21.7)	0.928
Weekdays	5.4 (3.1, 12.1)		5.1 (3.1, 12.1)	5.1 (3.1, 10.1)	7.1 (4.1, 15.1)	0.076	5.2 (3.1, 12.1)	5.8 (3.1, 12.1)	0.859
Weekends	5.5 (3.1, 9.1)		5.1 (3.1, 10.1)	5.1 (2.9, 9.1)	6.1 (3.1, 8.9)	0.611	5.1 (3.1, 8.2)	6.1 (3.1, 9.2)	0.665
**Physical activity in Median (25%, 75%)**									
Total MET-minutes	570 (132, 1220)		638 (120, 1295)	450 (173, 1139)	388 (75, 975)	0.201	598 (194, 1295)	353 (74, 1006)	0.028
Total minutes/week	130 (40, 250)		145 (40, 280)	145 (60, 280)	120 (30, 210)	0.214	150 (60, 280)	100 (30, 235)	0.032
Moderate	10 (0, 60)		5 (0, 60)	10 (0, 60)	10 (0, 60)	0.998	20 (0, 70)	0 (0, 50)	0.057
Strenuous	0 (0, 60)		0 (0, 90)	0 (0, 60)	0, (0, 40)	0.218	0 (0, 60)	0 (0, 60)	0.334
Walking	60 (20, 120)		60 (20, 150)	80 (20, 140)	60 (10, 100)	0.232	80 (30, 140)	60 (20, 100)	0.043
**Body Mass Index**	29.5 (25.9, 33.3)		28.9 (25.4, 33.7)	30.2 (26.6, 33.5)	29.3 (26.6, 33.1)	0.388	26.3 (24.1, 28.1)	33.7 (31.9, 37.3)	–
**At risk for lifestyle behaviors n (%)**									
>2 hours/day of TV∓		146 (43)	55 (44)	43 (37)	48 (48)	0.260	76 (43)	70 (43)	0.962
<150 min MVPA/week∓	163 (53)		53 (48)	53 (53)	57 (60)	0.238	79 (50)	84 (57)	0.260
BMI ≥30∓	218 (47)		92 (55)	78 (49)	80 (56)	0.397			

All estimates are reported in median (25% confidence limit, 75% confidence limit);

TV = television or video viewing; MET-minutes = metabolic equivalents of walking, moderate, and vigorous activities.

∓ Sample sizes may differ due to missing data. P-values are based on non-parametric Kruskal-Wallis test (for age group) and Wilcoxon rank-sum test (for obesity). Estimate for moderate, strenuous, and walking are expressed in minutes/week.

### Correlates of Lifestyle Behaviors

Bivariate associations among the medical, demographic, and lifestyle characteristics are reported in Table 3. In the following subsections, we describe the correlates of PA, television viewing, and obesity. Only the bivariate analyses are reported in Table 3. Results of the stepwise logistic regression models were reported here in the text for simplicity and clarity.

**Table 3:** Crude associations between medical and sociodemographic variables with physical activity, television viewing, and obesity status.

	Physical Activity			Television Viewing			Obesity		
	<150 min/week	≥150 min/week	Crude	<2 hrs/day	≥2 hrs/day	Crude	BMI<30	BMI≥30	Crude
	N (%)	N (%)	OR (95% CI)	N (%)	N (%)	OR (95% CI)	N (%)	N (%)	OR (95% CI)
**Age group**									
<50	40	32	0.62 (0.36, 1.08)	36	38	0.84 (0.50, 1.42)	37	34	1.02 (0.65, 1.60)
50–59	33	33	0.75 (0.43, 1.33)	37	29	0.64 (0.37, 1.10)	31	37	1.32 (0.84, 2.08)
60	27	35	1	27	33	1	32	29	1
**Years out from diagnosis**									
0–2	31	28	0.61 (0.33, 1.12)	31	31	0.79 (0.45, 1.38)	27	35	1.58 (0.98, 2.56)
3–4	25	21	0.55 (0.29, 1.05)	26	19	0.60 (0.32, 1.11)	24	21	1.10 (0.66, 1.86)
5–9	22	18	0.57 (0.29, 1.11)	19	20	0.82 (0.43, 1.54)	19	20	1.27 (0.74, 2.18)
10+	22	33	1	24	30	1	30	24	1
**Stage at diagnosis**									
I	35	33	0.84 (0.44, 1.62)	67	29	0.80 (0.43, 1.49)	36	34	0.73 (0.43, 1.21)
II	45	45	0.89 (0.47, 1.65)	42	49	1.19 (0.66, 2.12)	45	41	0.71 (0.43, 1.17)
≥ III	19	22	1	21	21		19	25	1
**Highest Education**									
≤ high school	6	9	2.58 (0.98, 6.81)	9	7	0.94 (0.39, 2.28)	7	11	**2.22 (1.08, 4.58)**
Some College	37	44	**1.88 (1.05, 3.35)**	39	40	1.27 (0.73, 2.21)	37	43	1.51 (0.94, 2.43)
College Graduate	26	27	1.60 (0.85, 3.01)	24	31	1.61 (0.88, 2.95)	29	25	1.13 (0.68, 1.90)
Graduate Degree	31	20	1	28	22	1	27	21	1
**Yearly Household Income**									
<35,000	20	29	1.72 (0.88, 3.39)	23	29	1.77 (0.94, 3.35)	19	31	1.61 (0.94, 2.76)
$35,000–64,000	19	21	1.32 (0.65, 2.69)	17	21	1.64 (0.81, 3.32)	20	17	0.87 (0.48, 1.56)
$65,000–$79,000	17	14	0.98 (0.45, 2.12)	15	18	1.60 (0.77, 3.35)	20	11	0.56 (0.30, 1.07)
> = $80,000	43	36	1	44	32	1	41	41	1
**Marital Status**									
Married	24	17	1.55 (0.88, 2.72)	22	22	1.04 (0.62, 1.75)	19	24	0.81 (0.52, 1.25)
**Weight Status**									
Normal	23	14	0.55 (0.30, 1.03)	20	18	0.93 (0.52, 1.66)	-	-	-
Overweight	32	34	0.93 (0.56, 1.55)	32	34	1.04 (0.64, 1.70)	-	-	-
Obese	45	52	1	48	48	1	-	-	-
**Number of comorbidities**	-	-	**1.40 (1.10, 1.77)**	-	-	1.18 (0.95, 1.47)	-	-	**1.35 (1.12, 1.63)**
**Smokers**	27	33	0.74 (0.45, 1.21)	30	27	1.21 (0.75, 1.95)	75	69	1.34 (0.89, 2.02)
**Hormone replacement**	46	50	0.83 (0.53, 1.31)	52	44	1.36 (0.89, 2.10)	47	48	0.95 (0.66, 1.36)
**>2 hrs Television/day**	42	45	0.88 (0.56, 1.38)	-	-	-	43	43	1.01 (0.66, 1.55)
**<150 minutes of MVPA**	-	-	-	48	45	1.14 (0.72, 1.80)	50	43	1.30 (0.83, 2.04)

For purposes of clarity, this table only contains results from the bivariate logistic regression analysis. Please see the text for the odds ratios (OR) and 95% confidence intervals (CI).

#### Not meeting guidelines for PA

Bivariate analyses revealed that a larger number of comorbid conditions were significantly associated with not meeting the current guidelines for PA. Similarly, in bivariate analyses, a lower education level was significantly associated with not meeting the current guidelines for PA (all P<0.05). In stepwise logistic regression models, the number of comorbid conditions was significantly associated with not meeting current guidelines for PA (OR = 1.50; 95% CI = 1.11, 2.03). In the stepwise model, women who completed high school (OR = 4.03; 95% CI = 1.00, 16.27) and those with some college (OR = 2.04; 95% CI = 1.03, 4.04) were less likely to meet the current guidelines for PA than were women who had graduate degrees.

#### Excessive television viewing

Socio-demographic and medical characteristics were not significantly associated with a television-viewing time of more than 2 hours per day in bivariate or multivariable models.

#### Obesity status

The number of comorbid conditions and level of education were significantly associated with self-reported obesity in bivariate models (all P<0.05). In stepwise logistic regression models, the number of comorbid conditions (OR = 1.67; 95%CI = 1.22, 2.27) was associated with increased risk for obesity, and women who were recently diagnosed (i.e., within the past 2 years; OR = 2.80; 95% CI = 1.32, 5.95) were more likely to be obese than were women who had been diagnosed 10 years prior to study enrollment.

## Discussion

In this study, we found that almost half of the women were obese, did not meet the current guidelines for PA, and had comorbid conditions. Fewer than half watched television for more than 2 hours per day. Comorbid conditions were prevalent in older women and women who were obese. Interestingly, we observed few lifestyle differences between population subgroups, but obese women were less active than non-obese women. In addition, the number of comorbid conditions appeared to be an important correlate of being obese and not meeting the current guidelines for PA. Our preliminary data show that AA breast cancer survivors need lifestyle interventions to curb high rates of inactivity and obesity, particularly because they are often burdened by other comorbid conditions that render them vulnerable to inactivity and obesity.

Approximately 50% of the women in our study reported having high blood pressure, and another 20% and 30% reported having diabetes or high cholesterol levels, respectively, and these rates appeared to increase with age. The rates of high blood pressure and diabetes observed among the women in our study are similar to those reported in other studies of minority women [32], yet these rates are substantially higher than national estimates for other women [33]. These data add to the current literature that suggests AA breast cancer survivors have high rates of comorbid conditions [8]. The exceedingly high rates of comorbid conditions observed in women who were older and in those who were obese are alarming, especially because comorbid conditions advanced age, and obesity are associated with overall- and cancer-specific survival. In particular, Tammemagi et al. [8] found that comorbid conditions among AA survivors account for approximately half of the overall survival disparity between AA and non-Hispanic white breast cancer survivors. Lifestyle interventions that target obesity among older AA cancer survivors are needed to combat or manage the comorbid conditions experienced by this population [22], [23].

Nearly half the women in our study were obese. These rates of obesity are comparable to those reported in previous studies of AA breast cancer survivors [22] yet greater than national averages among healthy AA women [33]. We found that obesity was significantly associated with the time since diagnosis and the number of comorbid conditions in multivariate models. The associations observed among comorbid conditions and obesity are congruent with previous findings [34]; however, the reason why PA and at-risk television viewing were not significantly associated with obesity in our models is not clear. The variability in the range of PA and television-viewing time could have attenuated the associations. Alternatively, categorizing women as obese or non-obese prevented us from evaluating the range of risk along the BMI continuum (i.e., normal, overweight, obese, and morbidly obese). Future research should examine various lifestyle factors (including diet) across the range of body size categories.

Our data indicate that 53% of the women in our study did not meet the current guidelines for PA and that 43% of them watched television for more than 2 hours per day. The percentage of women who did not meet the current guidelines for PA was similar to the percentage of healthy AA women not meeting PA guidelines [32]. However, the levels of television viewing appeared to be lower than those reported in the American Time Use Survey [35]. In particular, recent population-based data showed that women in the United States watch on average 3.28 hours of television per day [35]. The participants in our study watched an average of 12.2 hours of television per week or 1.74 hours per day. We are uncertain why television viewing in this population was considerably lower than estimates from national data. Our sample could have been biased toward educated women who spend less time watching television or a proportion of the women in our study could have misinterpreted the questionnaire items and reported their average daily television-viewing time rather than a weekly total. Unfortunately, there is no way to objectively measure television viewing among adults that is not intrusive. Self-report instruments that encourage adults to report the television shows that they watch on a given day may shed some insight into the specific amount of time they spend watching television.

The correlates of PA and SB in AA cancer survivors are not well understood. Herein, we found that education and comorbid conditions were significantly associated with PA, but we did not find any correlates for SB. AA breast cancer survivors reported similar levels of SB regardless of age, education, or health status. Alternatively, psychosocial constructs may be better correlates of PA and SB than demographic and medical conditions. Research assessing these correlates among AA breast cancer survivors is greatly needed.

The results from this study provide important and unique information about a minority group of breast cancer survivors and the lifestyle characteristics of this population. There are, however, several limitations of this study that should be noted. Our study focused exclusively on AA breast cancer survivors and adding a comparison group (e.g., non-Hispanic white survivors) may have helped to rule out the potential for confounding. The sample of AA breast cancer survivors was relatively healthy and well educated, so the results may not be generalizable to other populations of AA breast cancer survivors. In addition, PA and SB were assessed using a self-reported measure; therefore, recall and reporting biases might have existed. Moreover, these data are cross-sectional and do not imply causal inference. Nonetheless, to our knowledge, this is one of the first studies to report on the sedentary habits of AA breast cancer survivors. Future studies should consider including a comparison condition to examine potential similarities and differences in study outcomes.

In conclusion, these data show that many AA breast cancer survivors are overweight and obese, have comorbid conditions that may increase their risk for premature mortality, and are physically inactive. AA breast cancer survivors urgently need interventions that shield them from high rates of obesity and comorbid conditions. Ideally, these interventions will be based on their personal preferences and promote lifelong skills that enable them to adopt and maintain a physically active lifestyle. Researchers or institutions who partner with local and national organizations such as the Sisters Network may be able to harness the support needed to develop interventions in which minority breast cancer survivors are receptive to participating. Intervention studies may help to improve survival outcomes in this population.
